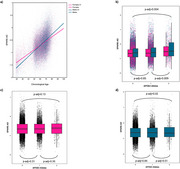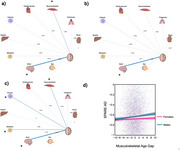# Sex‐specific patterns of a machine learning‐derived Alzheimer's brain atrophy imaging signature in participants without diagnosed cognitive impairment: A multi‐cohort study

**DOI:** 10.1002/alz70856_098833

**Published:** 2025-12-24

**Authors:** Filippos Anagnostakis, Mehrshad Saadatinia, Sarah Ko, Erdem Varol, Despina Kontos, Ajay Gupta, Adam Brickman, Li Shen, Guray Erus, Haochang Shou, Ye Ella Tian, Andrew Zalesky, Christos Davatzikos, Susan M. Resnick, Junhao Wen

**Affiliations:** ^1^ Laboratory of AI and Biomedical Science (LABS), Columbia University, New York, NY, USA; ^2^ Artificial Intelligence in Biomedical Imaging Laboratory (AIBIL), Center for and Data Science for Integrated Diagnostics (AI2D), Perelman School of Medicine, University of Pennsylvania, Philadelphia, PA, USA; ^3^ Tandon School of Engineering, New York University, New York, NY, USA; ^4^ Center for Innovation in Imaging Biomarkers and Integrated Diagnostics (CIMBID), Department of Radiology, Columbia University, New York, NY, USA; ^5^ Department of Radiology, Columbia University, New York, NY, USA; ^6^ Department of Neurology, Columbia University, New York, NY, USA; ^7^ Department of Biostatistics, Epidemiology, & Informatics, University of Pennsylvania, Philadelphia, PA, USA; ^8^ Artificial Intelligence in Biomedical Imaging Laboratory, Perelman School of Medicine, University of Pennsylvania, Philadelphia, PA, USA; ^9^ Centre for Biomedical Image Computing and Analytics, University of Pennsylvania, Philadelphia, PA, USA; ^10^ Melbourne Neuropsychiatry Centre, Department of Psychiatry, Melbourne Medical School, The University of Melbourne, Melbourne, VIC, Australia; ^11^ Laboratory of Behavioral Neuroscience, National Institute on Aging Intramural Research Program, National Institutes of Health, Baltimore, MD, USA; ^12^ New York Genome Center, New York, NY, USA

## Abstract

**Background:**

We investigated sex differences in a machine learning‐derived imaging signature of AD brain atrophy (i.e., SPARE‐AD^5^), in relation to age, genetic factors (*APOE* ε4 allele), and multi‐organ biological age gap (BAG^2,3^).

**Methods:**

Data from the iSTAGING and MULTI consortia included 53,622 participants without diagnosed cognitive impairment (mean age: 61.8 ± 12.6 years; 54% women). The SPARE‐AD model uses a support vector machine with a linear kernel to distinguish between cognitively normal individuals and those with AD^5^. Generalized linear models assessed sex differences and nine BAG associations with SPARE‐AD, adjusting for age, sex, APOE ε4, and interactions, and analysis of covariance (ANCOVA) with Tukey's test to assess differences in SPARE‐AD scores between *APOE* ε4 allele carrier groups.

**Results:**

Overall, SPARE‐AD increased with age (β = 0.018, *p* < 2e‐16). Women had higher SPARE‐AD scores than men (β = ‐0.393, *p* < 2e‐16). Women had higher SPARE‐AD scores at younger ages but lower values at older ages (β = 0.006, *p* < 2e‐16 for the age‐sex interaction term) when compared to males (Figure 1a). Furthermore, SPARE‐AD was positively associated with the number of *APOE* ε4 alleles (β = 0.018, *p* =  1.06e‐6). Non‐carriers and heterozygous carriers of the *APOE* ε4 allele exhibited lower SPARE‐AD scores compared to homozygous carriers in analyses of both combined sexes and in men alone; this pattern was not observed in women (Figure 1b‐d). Among the nine BAGs, the brain BAG was most strongly associated with SPARE‐AD in both sexes combined (β = 0.018, *p* =  1.09e‐302) (Figure 2a) and separately (women: β = 0.017, *p* =  5.08e‐128; men: β = 0.019, *p* =  4.24e‐175) (Figure 2b‐c). Other significant BAG associations were observed in men and not in women, including musculoskeletal (β = 0.004, *p* =  0.02), immune (β = 0.004, *p* =  0.02), and metabolic BAGs (β = 0.005, *p* =  0.02) (Figure 2b‐d).

**Conclusion:**

SPARE‐AD scores increased with age and were higher in women at younger ages but lower than men at older ages, with a significant age*sex interaction, and were positively associated with the number of the APOE ε4 allele, particularly in men.